# The Mfd protein is the transcription-repair coupling factor (TRCF) in *Mycobacterium smegmatis*

**DOI:** 10.1016/j.jbc.2023.103009

**Published:** 2023-02-11

**Authors:** Ogun Adebali, Yanyan Yang, Pradeep Neupane, Nneka I. Dike, Julia L. Boltz, Cansu Kose, Miriam Braunstein, Christopher P. Selby, Aziz Sancar, Laura A. Lindsey-Boltz

**Affiliations:** 1 Faculty of Engineering and Natural Sciences, Sabanci University, Istanbul, Türkiye; 2 Department of Computational Science - Biological Sciences, TÜBİTAK Research Institute for Fundamental Sciences, Gebze, Türkiye; 3 Department of Biochemistry and Biophysics, University of North Carolina-Chapel Hill, Chapel Hill, North Carolina, USA; 4 Department of Microbiology and Immunology, University of North Carolina-Chapel Hill, Chapel Hill, North Carolina, USA

**Keywords:** nucleotide excision repair, transcription-coupled repair, excision repair-sequencing, UvrD, tuberculosis, CPD, cyclobutane pyrimidine dimer, NCBI, National Center for Biotechnology Information, NER, nucleotide excision repair, NTS, nontranscribed strand, qRT–PCR, quantitative RT–PCR, RNAP, RNA polymerase, TCR, transcription-coupled DNA repair, TRCF, transcription-repair coupling factor, TS, transcribed strand, XR-Seq, excision repair-sequencing

## Abstract

*In vitro* and *in vivo* experiments with *Escherichia coli* have shown that the Mfd translocase is responsible for transcription-coupled repair, a subpathway of nucleotide excision repair involving the faster rate of repair of the transcribed strand than the nontranscribed strand. Even though the *mfd* gene is conserved in all bacterial lineages, there is only limited information on whether it performs the same function in other bacterial species. Here, by genome scale analysis of repair of UV-induced cyclobutane pyrimidine dimers, we find that the Mfd protein is the transcription-repair coupling factor in *Mycobacterium smegmatis*. This finding, combined with the inverted strandedness of UV-induced mutations in WT and *mfd*^-^*E. coli* and *Bacillus subtilis* indicate that the Mfd protein is the universal transcription-repair coupling factor in bacteria.

To quote a review by Portman and Strick (1), “Transcription-coupled DNA repair (TCR), a subpathway of nucleotide excision repair (NER) … has a specific meaning with specific consequences… One hallmark of this process is that it is the ubiquitous transcribing RNA polymerase (RNAP) which initially detects the DNA damage by stalling when it encounters the lesion on its transcribed strand…a second hallmark of TCR is that, by coupling repair to polar DNA transcription, it leads to enhanced repair rates of lesions on the transcribed strand relative to those lesions located on the nontranscribed strand. The protein responsible for coupling transcription to enhanced repair of the transcribed strand in *Escherichia coli* is Mfd,” also called TRCF (transcription-repair coupling factor) (1, 2, 3, 4, 5, 6, 7). A third hallmark of TCR is that ablation of the *mfd* gene increases the mutation frequency of UV-induced mutations in *E. coli* held in nongrowth medium prior to plating on selective media (8). Finally, while UV-induced mutations in WT *E. coli* or *Bacillus subtilis* (*B. subtilis*) are mostly caused by damage in the nontranscribed strand (NTS), in the *mfd*^*-*^ mutant strain, most of the UV-induced mutations are caused by damage in the transcribed strand (TS) such that the *mfd*^*-*^ mutation leads to ∼14-fold change of mutagenic lesion being in the NTS to being in the TS (9, 10).

At the biochemical level, TCR has been reconstituted *in vitro* with five *E. coli* proteins, UvrA, UvrB, UvrC, UvrD, and Mfd along with RNAP, the four rNTPs, and an appropriate DNA transcription–repair substrate (5). Studies with this system have shown that Mfd displaces RNAP stalled at a lesion site (UV, cisplatin, and psoralen) and simultaneously recruits UvrA_2_B to the damage by its UvrA-binding domain and in so doing accelerates the rate of repair threefold to fivefold (4, 11). These *in vitro* findings have been confirmed by ensemble biochemical experiments (6, 12, 13, 14, 15, 16), structural analyses (17, 18, 19, 20, 21), as well as *in vitro* (22, 23) and *in vivo* (24, 25, 26) single-molecule analyses, and genomics (27, 28).

However, even though the *mfd* gene is universally found in all bacterial species analyzed, its biochemical function, other than *E. coli*, has been analyzed only in *B. subtilis* where, as in *E. coli*, it was found that it releases RNAP stalled at a psoralen-thymine roadblock, along with the truncated transcript; but the subsequent repair reaction was not investigated in this study (9). In recent years, most of the work on Mfd has focused on its role in resolving replication–transcription conflicts (16, 29) and removing intragenic repressor protein or secondary structure transcription blocks (7, 30, 31, 32, 33, 34) and in so doing giving rise to transcriptional regulation and appearance of mutants that confer resistance to various antibacterial agents; and hence Mfd has been called an “evolvability factor.” However, there has been no report that Mfd mediates TCR in bacteria other than *E. coli*. Hence, we wished to investigate the role of Mfd in TCR in Mycobacteria because recently there has been great interest in the role of Mfd as an evolvability factor in *Mycobacterium tuberculosis*. In our study, we used the nonpathogenic *Mycobacterium smegmatis* to determine whether Mfd is the TRCF in this genus. Our analysis of UV damage repair in various mutants of *M. smegmatis* lead us to conclude that Mfd is the TRCF in Mycobacterium as it is in *E. coli*.

## Results

### Ablation of mfd in *M. smegmatis*

**Figure 1:**
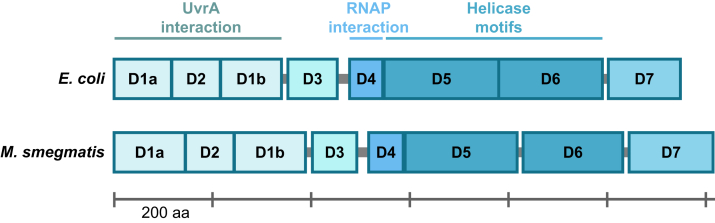
**The domain structure of Mfd.** The modular Mfd protein consists of eight domains indicated as *colored boxes*: the N-terminal UvrA interaction region (D1a, D2, D1b), D3, D4 RNA polymerase (RNAP) interaction region, D5 and D6 helicase motifs, and the C-terminal D7 autoinhibitory domain. The domain assignments of the *Escherichia coli* and *Mycobacterium smegmatis* Mfd proteins are indicated as reported previously (20, 37).

We previously demonstrated that in *M. smegmatis* (35), as in *E. coli* (28), UV-induced cyclobutane pyrimidine dimers (CPDs) are excised in the form of 12 to 13-mers by nucleotide excision repair, and these excised oligonucleotides were isolated and mapped to the genome using the excision repair-sequencing (XR-Seq) method (36) to investigate repair patterns. As this method allows for the genome-wide analysis of TS/NTS repair ratios, we were able to clearly observe the presence of TCR in *M. smegmatis* (35). In *E. coli*, after employing this powerful method on different mutant strains, it was clear that Mfd is the TRCF in this bacterial lineage (27, 28), but the role of Mfd remains to be determined for other lineages. The *E. coli* and *M. smegmatis* Mfd proteins share 38% amino acid sequence identity and have similar domain organization (Fig. 1). The crystal structures for *E. coli* (20) and *M. smegmatis* (37) Mfd homologs have been solved, and both exhibit conservation of eight domains grouped into five functional modules (34): UvrA-interacting module (D1a–D2–D1b), domain D3, the RNAP-interacting domain D4, the ATP-dependent helicase module (D5–D6), and the C-terminal domain D7.

**Figure 2:**
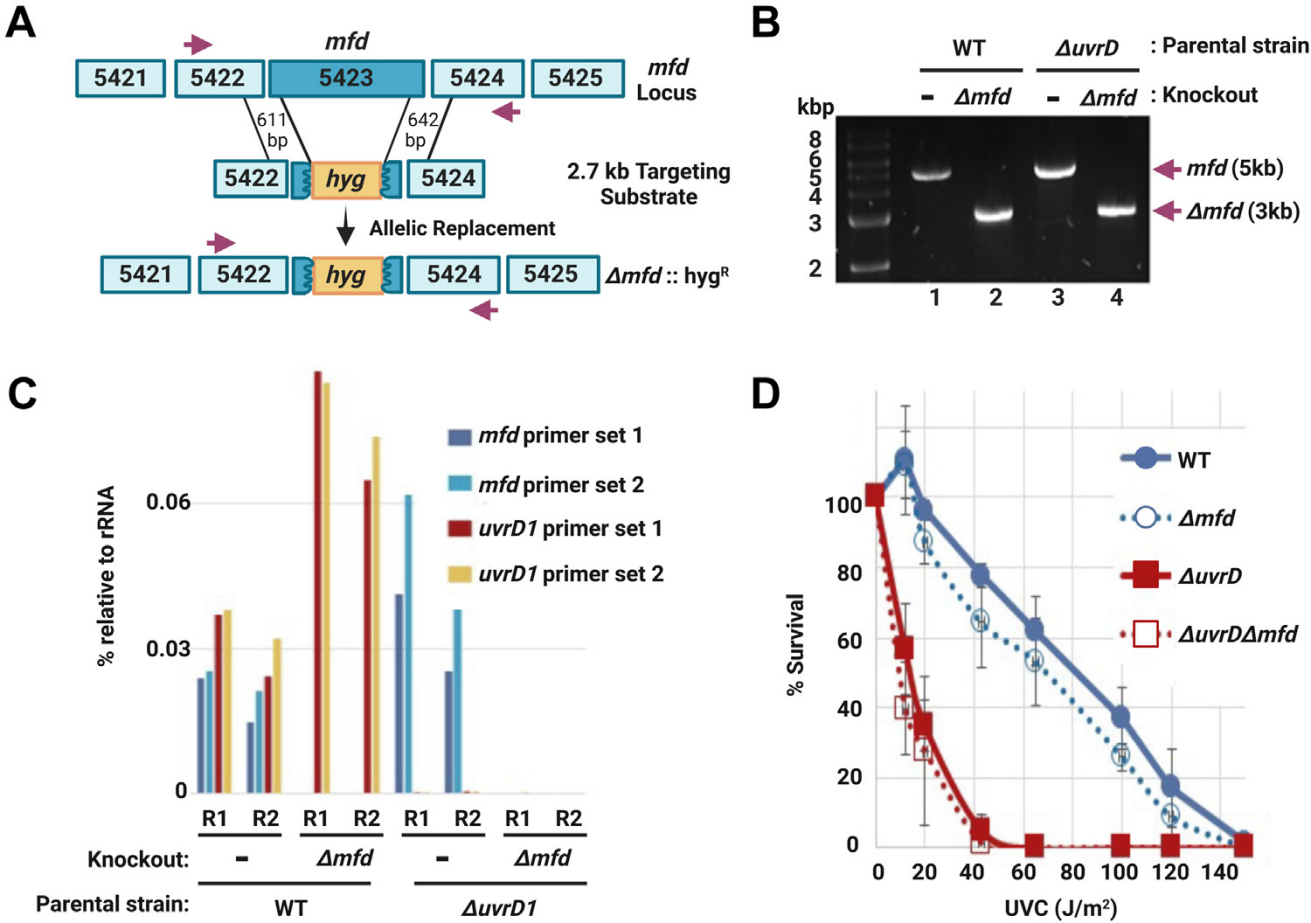
**Ablation of *mfd* in *Mycobacterium smegmatis*.** The diagram in *A* illustrates the recombineering knockout strategy. The *mfd* gene (MSMEG_5423) and two upstream and downstream genes are shown. The 2.7 kb targeting substrate contained sequences 611 bp upstream and 642 bp downstream of *mfd* flanking hygromycin (*hyg*) to confer hygromycin resistance after allelic replacement in the knockout strain (Δ*mfd*::hyg^R^). The primers used for PCR knockout confirmation are indicated by *purple arrows*. *B*, shows DNA products of the size expected (3 or 5 kb) from PCR amplification of the *mfd* locus in genomic DNA isolated from the four *M. smegmatis* strains, WT (WT, lane 1), Δ*mfd* (lane 2), Δ*uvrD* (lane 3), and Δ*uvrD*Δ*mfd* (lane 4). *C*, quantitative RT–PCR (qRT-PCR) was used to measure the mRNA abundance of *mfd* and *uvrD* relative to rRNA in the four *M. smegmatis* strains. Two different primer sets were used for each gene, and two biological repeats are shown (R1 and R2). *D*, the four *M. smegmatis* strains were tested for UV sensitivity as described under “Experimental procedures” section. The *mfd* knockout survival curves are not significantly different than their parental strains when analyzed by unpaired Student’s *t* test.

In order to analyze the role of Mfd in mycobacterial TCR, we deleted the *mfd* gene (MSMEI_5274) from both WT and *ΔuvrD1* (MSMEI_5382) (38) strains of *M. smegmatis* mc^2^155. The *ΔuvrD* mutant was used because in *E. coli* (27, 28, 39), and to a somewhat lesser degree in *M. smegmatis* (35), following excision the excised 12- to 13-mer is degraded rather rapidly by exonucleases reducing the yield of material needed for high coverage mapping, and in a *ΔuvrD* background, the excised oligomer remains genome bound and protected by UvrB–UvrC, thus enabling higher yields of full-length excised oligomers. In both *M. smegmatis* strains, the entire open reading frame of *mfd* was replaced with the *hyg*^*R*^ selection marker using standard recombineering allelic exchange procedures (40). PCR amplification of the predicted fragment sizes from genomic DNA (Fig. 2*A*) confirm targeted insertion at the *mfd* locus in both strains (Fig. 2*B*). To further confirm that there is no expression of the *mfd* and *uvrD1* genes in their respective knockout strains, we performed quantitative RT–PCR (qRT–PCR) (Fig. 2*C*). We did not observe any obvious growth defects between the parental strains and their corresponding *mfd* knockouts in liquid culture or on agar medium. We tested the four *M. smegmatis* strains for sensitivity to UV irradiation, and as previously observed in *E. coli*, deletion of the *mfd* gene does not result in very pronounced UV sensitivity when compared with the parental strains (Fig. 2*D*). We did confirm that the *ΔuvrD1* strain is extremely UV sensitive as has been previously reported (38). The relative UV sensitivities are consistent with the roles of UvrD and Mfd and the consequences of their deletion in *E. coli*; catalytic turnover of excision repair proteins is abolished in the *uvrD* mutant, but repair is merely delayed in the *mfd* mutant.

### XR-Seq analysis of nucleotide excision repair events in Δmfd *M. smegmatis*

**Figure 3:**
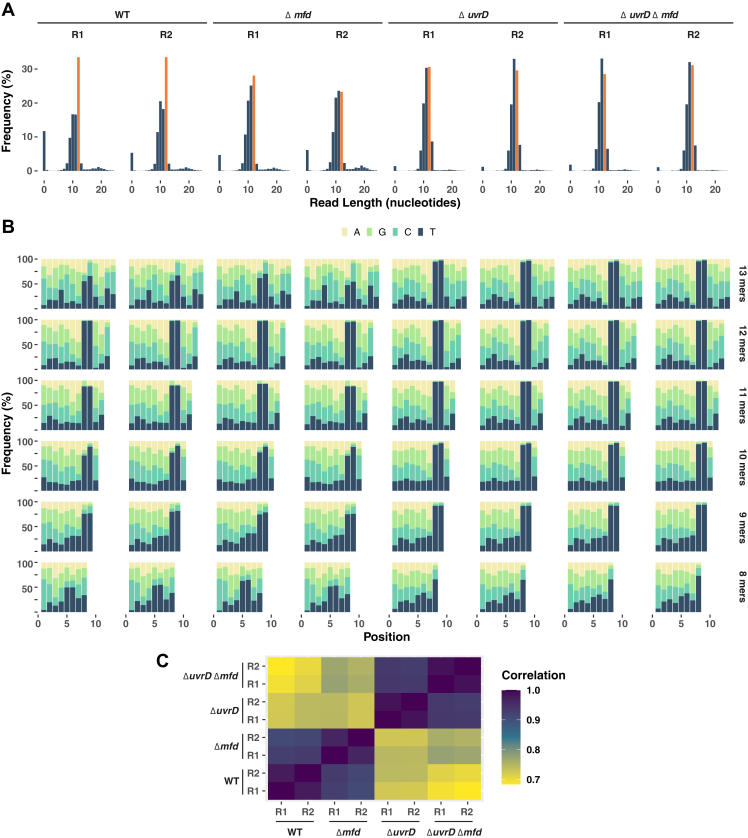
**Excision repair-sequencing (XR-Seq) analysis of excision sites in *Mycobacterium smegmatis*.** WT or Δ*uvrD* mutant cells and their *mfd* knockout derivatives were irradiated with 100 Jm^−2^ of 254 nm UV and incubated at room temperature for 5 min before isolation of the cyclobutane pyrimidine dimer (CPD)-containing excised oligonucleotides. The read-length frequency distribution histograms (*A*) for both repeats (R1 and R2) in all four strains show that the predominant excision product size is 12-nt (*orange*), and there is a higher proportion of 13-nt excision products in the Δ*uvrD* mutant strains relative to the WT strains. The histograms in (*B*) are aligned with the samples in panel (*A*) above and show the nucleotide frequency (*y*-axis) at each position (*x*-axis) of the 8- to 13-nt excision products. Reads were plotted by locating the 5′ residue at position 1. Notably, the position of the dipyrimidine peak (presumptive CPD damage site) in the excision products is consistent with the formation of the 5′ incision 7 nt from the CPD, and the 3′ incision 4 nt from the CPD in the 13-mer, and consistent with degradation occurring from the 3′ end in the smaller captured excised oligos. Correlation coefficients of the normalized total repair levels for the genes between each sample (including replicates R1 and R2) are shown in (*C*).

We performed XR-Seq analysis on excision products isolated from the four strains of *M. smegmatis* following irradiation with UV. In this procedure, DNA fragments isolated from treated cells are ligated to adapters, photoreactivated to repair the CPDs, amplified by PCR, sequenced by next-generation sequencing, and reads are mapped to the genome (41). In Figure 3*A*, the read lengths are plotted to show the read length as a function of their frequency. The prevalent reads are 11- to 13-mers in all strains. The quality of the reads was further evaluated by plotting the nucleotide distribution along the length of the excision fragments (Fig. 3*B*). As apparent from full-length excision products, positions 8 to 9 nucleotides from the 5′ end and 4 to 5 nucleotides from the 3′ end contain almost exclusively T-T (or more precisely Pyr-Pyr) dinucleotides, consistent with the prokaryotic pattern of dual incisions 7 nucleotides 5′ and 3 to 4 nucleotides 3′ to the Pyr-Pyr. Correlation coefficients of the normalized total repair levels for the genes between each sample can be seen in Figure 3*C*. We observe that very high correlation between the replicates and the parental strains have higher correlation with their *mfd* knockouts than between the two parental strains.

### Mfd is required for TCR in *M. smegmatis*

**Figure 4:**
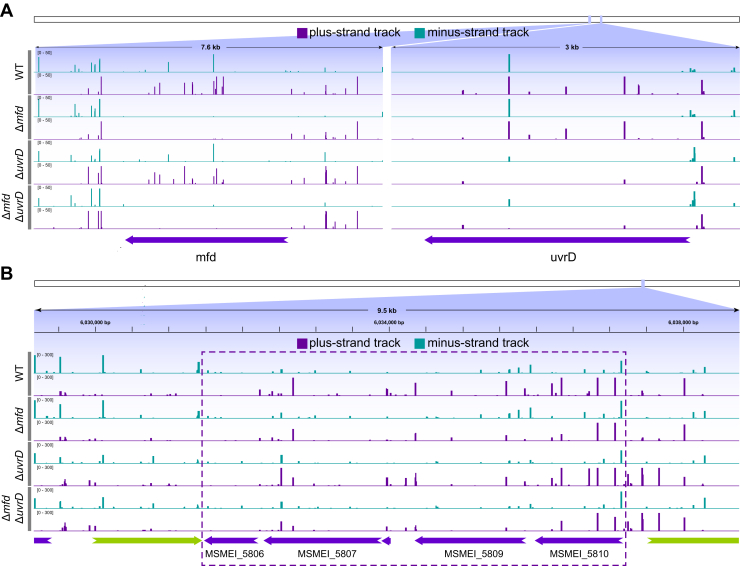
**Transcription-coupled repair in *Mycobacterium smegmatis*.***A*, screenshots show the repair profiles of plus and minus strands in *purple* and *green*, respectively for the four strains in the genomic regions of the knocked-out genes, *mfd* (*left*) and the *uvrD* (*right*). Both these genes (indicated by the *purple arrow-headed bars*) are located on the minus strand, and thus the transcribed strand (TS) repair tracks are *purple* and nontranscribed strand (NTS) repair tracks are *green*. TS/NTS ratios for the *mfd* gene for the WT and Δ*uvrD* strains are 1.13 and 1.71, respectively; and for the *uvrD* gene, the TS/NTS ratios for the WT and Δ*mfd* strains are 2.30 and 1.47, respectively. *B*, a screenshot panel illustrates repair in a representative ∼10 kbp region of the *M. smegmatis* genome for the four strains. The *arrow-headed bars* (colored in *purple* and *green*) at the *bottom* of the tracks indicate genes and their direction. As in (*A*), for the genes on the minus strand (*purple arrow-headed bars*), their transcribed strand (TS) repair tracks are *purple* and their nontranscribed strand (NTS) repair tracks are *green*. The two biological replicates for each strain are merged, and repair is plotted as reads per million total reads (RPM) with the scale from 0 to 300. Based on the read counts in the interval of the potentially polycistronic operon between 6,031,500 and 6,037,200 (*dashed box*), TS/NTS ratios for each strain are the following: WT: 2.53; the Δ*mfd*: 0.97; Δ*uvrD*: 3.23; Δ*uvrD* Δ*mfd*:1.33.

We used our XR-Seq data to analyze TCR in the four *M. smegmatis* strains. The excised oligonucleotide reads were mapped to the genome and assigned to the TS or NTS based on the publicly available *M. smegmatis* transcriptome (42). As is the case in *E. coli*, the vast majority of the *M. smegmatis* genome is transcribed with frequent same-direction transcriptional overlap and antisense transcription. Even with these caveats, there is still a TS-bias of repair genomewide in WT and *ΔuvrD* mutant strains that is abrogated by the deletion of *mfd*. Figure 4*A* shows screenshots illustrating levels of repair in the four *M. smegmatis* strains in the region of the genome containing the knocked-out *mfd* and *uvrD1* genes. Repair in both strands is shown, and for both genes, the TS is the + strand (repair in *purple*). The results illustrate the loss of repair signal within the regions deleted. The results also suggest modest TCR in the *uvrD* gene, as indicated by a reduced TS/NTS repair ratio in the *mfd* mutant strain compared with WT. Unfortunately, the apparent TCR in the uvrD gene is not definitive because of the small gene size and weak repair signal (*y*-axis = 0–50 reads). Figure 4*B* shows a screenshot of repair within a 9.5 kb region of the *M. smegmatis* genome, which includes a polycistronic operon (in the *boxed area*) where a stronger repair signal is seen (*y*-axis = 0–300). Here, the TS is the + strand (repair in *purple*). Visual inspection suggests TCR in the WT and Δ*uvrD* strains, which is abrogated by deletion of *mfd*. The normalized TS/NTS ratios for this region are 2.53 in WT; 3.23 in Δ*uvrD*; 0.97 in Δ*mfd*; and 1.33 in Δ*uvrD*Δ*mfd*.

**Figure 5:**
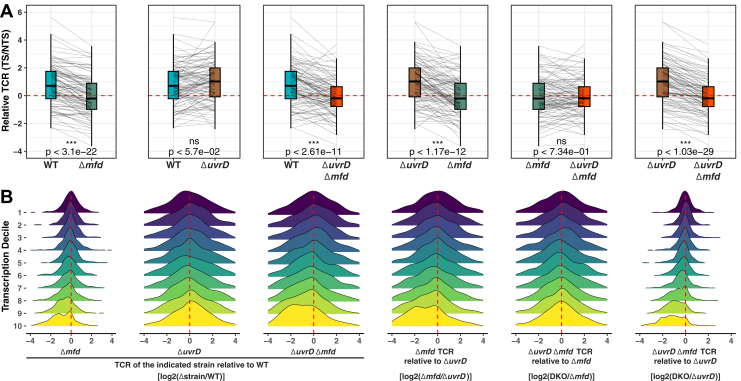
**Transcription-coupled repair depends on Mfd in *Mycobacterium smegmatis.****A*, pairwise normalized transcribed strand (TS)/nontranscribed strand (NTS) level comparison of genes in the top quartile of gene expression. Paired *t* test (*p* values) indicate the significance of the mean difference between the two strains. Replicate 1 data set was used. *B*, density plots of TS/NTS difference between strains for each transcription decile group of genes. Transcription increases from decile 1 (lowest) to 10 (highest).

The XR-Seq data were then analyzed to assess repair among all genes. Figure 5*A* illustrates TS/NTS repair ratios for *M. smegmatis* genes with the highest quartile of gene expression. The different plots compare each possible strain combination. For WT and Δ*uvrD* mutant strains, the positive TS/NTS values are indicative of TCR, which is absent in both *Δmfd* strains where the log2-transformed TS/NTS ratios shift more to the negative values. The preferential repair of the NTS in the absence of Mfd is due to blocking of TS repair by stalled RNAP (43). TCR appears essentially the same in WT and *ΔuvrD* mutant cells and is absent when there is no Mfd protein, which was also observed in *E. coli* and likely occurs in all prokaryotes that possess full-length Mfd. Paired *t* test analyses for each pair show that the decrease in TS/NTS values in the absence of Mfd compared with its presence in both WT and Δ*uvrD* strains is significant. However, there is no significant TCR level difference in the presence and absence of UvrD in both WT and Δ*mfd* strains. The average log2(TS/NTS) level that is over 1 in Δ*uvrD* mutant is observed to decrease to below 0 in both Δ*mfd* and double knockout Δ*uvrD*Δ*mfd* strains.

Prior findings in *E. coli* and other organisms have shown an association between transcription and TCR levels (27, 28, 44, 45), so we analyzed our XR-Seq data in more detail to see whether this was also the case in *M. smegmatis*. Figure 5*B* shows density plots of log_2_-transformed TS/NTS ratio differences between each strain pair combination, grouped into deciles of gene expression (where 10 is highest expression). Here, the data show a strong effect of transcription level on TCR. The log2-transformed TS/NTS ratio of one strain divided by another (Fig. 5*B*) is negative when the strain in the numerator has the *mfd* mutation, and this effect is most pronounced at high transcription levels. In contrast, there is essentially no difference between TCR levels for genes in low deciles that are not expressed.

## Discussion and conclusion

Here, we have demonstrated that mycobacterial Mfd, as in *E. coli*, is the TRCF for repair of UV-induced DNA damage. The *mfd* gene was identified as being responsible for the phenomenon (discovered in 1956) of reduced frequency of UV-induced mutations when *E. coli* cells were held briefly in nongrowth medium before plating on selective media (8). TCR in *E. coli* was discovered in 1989 (2), and in 1991, it was demonstrated *in vitro* that the *mfd* gene product was essential for TCR, and therefore, the protein was named TRCF (4). In subsequent years, the Mfd protein was characterized in great detail by biochemical (3, 5, 46), crystallographic (20), cryo-EM (21, 47), and *in vitro* and *in vivo* single-molecule (22, 23, 24, 25, 26, 29, 47, 48) analyses to arrive at a comprehensive and internally consistent model for how TCR works in *E. coli*, and by extension in all bacterial species that express Mfd (1, 12, 33, 34, 49).

Subsequent to the discovery of Mfd as the *E. coli* TRCF, *B. subtilis* Mfd was shown to have properties similar to *E. coli* Mfd, and moreover, it was shown to play a role in recombination and stationary phase mutagenesis (9). More recently, there has been renewed interest in Mfd as the protein involved in double-strand breaks arising from R-loops during transcription and from transcription-replication fork collisions, and interest in the role of Mfd in gene regulation by dissociating RNAP impeded by intragenic protein complexes or hard-to-transcribe DNA secondary structures (16, 29, 30, 31). These forms of DNA have been proposed to give rise to mutations that confer resistance to a number of drugs in *B. subtilis* and importantly in *M. tuberculosis* by a phenomenon named “evolvability factor” (31, 32). Consequently, there is great interest currently in Mfd as a target for new antimicrobials for tuberculosis that has developed resistance to standard antituberculosis drugs. Thus, *mfd*, which was a relatively obscure gene with a somewhat exotic effect on UV-induced mutagenesis (8), appears to be the universal TRCF in bacteria but at the same time a multifunction translocase (13, 14, 15, 16) with practical medical applications.

## Experimental procedures

### Recombineering and knockout confirmation

The *M. smegmatis Δmfd* mutant strains were created using the recombineering strategy described previously (50) and outlined in Figure 2*A*. A recombineering plasmid (pUC19-MfdHyg) was generated with primers described in Table S1. Briefly, the Gibson Assembly Protocol (New England Biolabs; E5510) was used to generate pUC19-Mfd containing a unique EcoRV site between the ∼600 base pair upstream and downstream flanks of *mfd*. A hygromycin-resistance (hyg^R^) cassette was removed from pMP1064 (51) with SmaI and cloned into the EcoRV site of pUC19-Mfd to generate pUC19-MfdHyg, which was then digested with XbaI and SphI to release a 2740 base pair linear fragment containing the upstream flank of *mfd*, the hyg^R^ cassette, and downstream flank of *mfd*. This DNA fragment was transformed *via* electroporation into both WT (mc^2^155) and *ΔuvrD1* strains containing the recombinase plasmid pJV53 (40) conferring kanamycin resistance. Double crossover allelic exchange recombinants were selected for by resistance to hygromycin, and strains were cured of pJV53 by passaging three times in the absence of kanamycin. PCR was performed with primers shown in Table S1 with purified genomic DNA QIAamp DNA Mini Kit (QIAGEN) to screen for knockouts as shown in Figure 2*B* and were confirmed with Sanger Sequencing (Genewiz from Azenta). *M. smegmatis* were grown with appropriate antibiotics for plasmid selection (50 μg/ml hygromycin or 20 μg/ml kanamycin).

#### qRT–PCR analysis

Total RNA was isolated from *M. smegmatis* cultures using a PureLink RNA Mini Kit (Thermo Fisher Scientific), and complementary DNA was synthesized from 50 ng of RNA using iScript cDNA Synthesis Kit (Bio-Rad). qRT–PCR was carried out in triplicate on a QuantStudio Real-Time PCR System (Life Technologies) with iTaq Universal SYBR Green Supermix (Bio-Rad) and the primers listed in Table S1. Analysis of qRT–PCR data was carried out using standard comparative CT methods and normalizing to 16S rRNA amplified from the same sample. The data presented are two independent experiments.

#### UV sensitivity analysis

The four *M. smegmatis* strains were grown to an absorbance of ∼0.3 at 600 nm, diluted 10^−^^6^ in LB, and then 0.1 ml was plated on LB agar plates (∼200 colony-forming units). The plates were irradiated with the indicated UVC doses using a 254 nm UV light source and incubated in the dark for 3 days at 37 °C. The surviving colonies were counted and normalized to the colony counts from unirradiated plates. The data presented are averages of three independent experiments, and error bars represent standard error of the mean.

### XR-Seq assay

*M. smegmatis* cultures were grown, and XR-Seq samples were prepared as described previously, using the anti-CPD immunoprecipitation steps to isolate excision products bearing CPD damage (35) with the following modifications. Approximately 150 ml of cells that underwent 5 min repair were used for XR-Seq, and the XR-Seq libraries were prepared using the A3b 3′-adapter containing an 8-base random sequence (27) to provide a unique molecular identifier to allow the identification and elimination of duplicate reads introduced in the subsequent PCR step.

### Data analysis

The data analysis workflow is shown in Fig. S1 and described later.

#### Removal of PCR duplicates

We prepared excision-repair sequencing in two biological replicates for each strain of WT, *Δ*mfd, *Δ*uvrD, and double knockout (*Δ*uvrD*Δ*mfd). Because we used unique molecular identifiers consisting of eight random bases, we could remove PCR duplicates *via* a custom script (see the repository).

#### Adaptor trimming

After removing PCR duplicates, we trimmed the adaptor from the 3′ end. We used cutadapt, version 3.4 (https://journal.embnet.org/index.php/embnetjournal/article/view/200) (52) by allowing flexibility in the unique molecular identifier adaptor region with the following sequence: GGCTCAGTTCGTATGAGTGCCGNNNNNNNN. We discarded the reads that do not contain any adaptor using the parameter “--discard-untrimmed.”

#### Quality control for XR-Seq samples

We retrieved the length distribution of the excised oligomers with a custom script (see the repository). We also calculated the nucleotide distribution of the most abundant size of the excised products with a custom script (see our repository).

#### Alignment

We aligned reads on the *M. smegmatis* str. MC2 155, complete reference genome (CP001663.1) using Bowtie2 (version 2.4.5) with the following seed parameter “--seed 1.” We have downloaded the fasta file from National Center for Biotechnology Information (NCBI) (https://www.ncbi.nlm.nih.gov/nuccore/CP001663.1?report=fasta). We created the Bowtie2 index files with the bowtie2-build command (53, 54).

#### Aligned read filtering

Because of the length of the excised oligomers (mainly 10–13 nucleotides), and the size of the genome, it is possible that some individual reads may map to multiple sites in the genome. To exclude the ambiguity of multimapping reads, we generated “singleton unique damage sites” using the reference genome for 10 to 13-mer DNA regions requiring dithymines at the eighth and ninth positions from the 5′ site. We realized that the number of singleton 10 and 11-mer damage sites is expectedly small throughout the genome. To avoid a bias of heterogeneous distribution of short theoretical singletons throughout the genome, we continued the analysis with 12- and 13-mer reads. Furthermore, because the number of 13-mer oligomers is too low and the singleton unique 13-mer theoretical position number is high, our samples do not cover most of the possible 13-mer positions. To avoid unequal sampling of potential excision sites, we trimmed 13-mer reads by 1 nucleotide from the 3′ end and treated them as 12-mer reads. In the final stage, our singleton list consisted of 12-mer singletons only.

#### Genome coverage files

First, the aligned reads in the bed files are separated into plus and minus strands using an awk command (see the repository). We converted the strand-separated bed files to bedgraph format using “bedtools genomecov” with the following parameters -bg -scale N (55). N denotes the total number of reads in the bed file. The generated bedgraph file is converted to bigwig with the ucsc-bedGraphToBigWig (version 377) tool. The bedtools version used in this step was 2.29.0. The bigwig files were visualized using IGV (http://software.broadinstitute.org/software/igv/, Broad Institute, and the Regents of the University of California; (56, 57)).

#### Domain architecture of the Mfd protein

Custom javascript code was used to generate the to-scale domain architecture of the Mfd proteins (Fig. 1).

#### Simulation for the expected read count per gene

Some genes harbor more potential excision sites than others. This is also true for the strands: TS and NTS. To eliminate the effect of sequence context differences between genes and between strands, we performed an excision repair simulation. We randomly generated the N number of reads per each sample. N is the total number of the mapped reads for each sample. We used a custom script for this process (see the repository). Although there are 105 K unique potential 12-mer excision sites, not all the potential damage sites were covered in the data sets. We called these “unrepairable sites” where we never observed an excised oligomer. For this reason, we only used the unique 12-mer potential excision sites observed in at least a single biological replicate of any strain.

#### Strand-based read count per gene

For simulation and XR-Seq data, we counted the reads mapped onto the TS and NTS of each gene. We retrieved the gene list from gtf file downloaded from NCBI (https://www.ncbi.nlm.nih.gov/nuccore/CP001663.1). We converted the gtf file to a bed file using a custom script. To count the repair events for each gene, we used bedtools intersect with the following commands -S and -s for TS and NTS, respectively.

#### Sample correlation

The correlation between eight samples was analyzed using the overall repair per gene. The Pearson's correlation coefficient was calculated in R.

#### RNA-Seq pipeline

Two RNA-Seq paired-end samples (Sequence Read Archive IDs: SRR5983958 and SRR5983959) were retrieved using fasterq-dump command in the sra-tools package (version 3.0.0) *via* a parameter --skip-technical. The adaptor trimming was performed with trimmomatic as originally applied with the following parameter: “TRAILING:3.” The alignment was performed with Bowtie2 with the default parameters. The gene-based RNA-Seq read counts were obtained as in the XR-Seq analysis. Reads per kilobase per million mapped reads values were calculated in R. Total mapped reads were calculated using the reads mapping onto the entire gene set. Intergenic reads were not considered for this metric. We used two biological replicates separately to obtain top-quartile genes. The genes that appeared to be in the first quartile for both replicates were used as consensus Q1 genes (Fig. 5*A*).

#### Analysis of the TS/NTS values with the high-quality genes

Since genes with low numbers of observed or expected repair events would create noise in normalized (observed/expected) TS/NTS ratios, we applied a filter and required genes to have at least 10 total observed or expected reads in any of the eight samples (four strains with two replicates). We also further selected the genes with at least five mappable repair sites from each TS and NTS strand in all samples and excluded the other genes. These filtering steps resulted in 109 genes used in Figure 5*A*. Because the biological XR-Seq replicates are highly correlated with each other, we merged biological replicates for this analysis. The paired *t* test was applied to test the significance of the mean differences between strains. For transcription decile categorization (Fig. 5*B*), we filtered out the genes with varying transcription levels by requiring retained genes in two replicates to be categorized in the same transcription decile group from 1 (low) to 10 (high).

#### Computing resources and reproducibility

The data analysis part of this project is fully reproducible. We used snakemake workflow (58) to allow scalability and reproducibility. All the custom scripts and commands, and parameters for the publicly available tools, can be found in the repository. We used computing clusters at Sabanci University to perform the data analyses. The cluster had Slurm workload manager, and by default, the workflow was configured to work in clusters with Slurm. However, Snakemake allows running the workflow on any Linux-based server.

## Data availability

The raw data have been deposited in the Sequence Read Archive of the NCBI under accession number PRJNA912722. The codes can be accessed in this repository: https://github.com/CompGenomeLab/MycoSmegmatis_TRCF.

## Supporting information

This article contains supporting information.

## Conflict of interest

The authors declare that they have no conflicts of interest with the contents of this article.
