# Supplementary material for: The Mfd protein is the transcription-repair coupling factor (TRCF) in Mycobacterium smegmatis
Source: J Biol Chem. 2023 Feb 11;299(3):103009. doi: 10.1016/j.jbc.2023.103009 (PMC10023983; doi:10.1016/j.jbc.2023.103009)
Supplement: Supplemental data [file mmc1.pdf]

Supplemental Material

The Mfd protein is the Transcription-Repair Coupling Factor (TRCF) in *Mycobacterium smegmatis*

Ogun Adebali[1,2], Yanyan Yang[3], Pradeep Neupane[4], Nneka I. Dike[3], Julia L. Boltz[3], Cansu Kose[3], Miriam Braunstein[4], Christopher P. Selby[3], Aziz Sancar[3,*], and Laura A. Lindsey-Boltz[3]

[1]Faculty of Engineering and Natural Sciences, Sabanci University, Turkey 34956; [2]TÜBİTAK Research Institute for Fundamental Sciences, Gebze, Turkey 41470 [3]Department of Biochemistry and Biophysics, University of North Carolina-Chapel Hill, Chapel Hill, NC 27599; and [4]Department of Microbiology and Immunology, University of North Carolina-Chapel Hill, Chapel Hill, NC 27599

Primer Name	Purpose	Sequence (5'-3')	Description
5'Mfd_fwdNEB	Gibson cloning with BamHI-digested pUC19 to generate pUC19-Mfd. A Hyg-containing cassette (SmaI) from pMP1064 was cloned into the EcoRV site of pUC19-Mfd to generate pUC19-MfdHyg	CTCGGTACCCGGGGATCCTCTAGA GTCGATAGAAGAGGTCGCCCAA	For PCR amplification upstream of *mfd* (MSMEG_5423)
5'Mfd_revNEB		GCTTCGCCTCGATATCGGTCATGAT GTGTCCATCTTAGGCG	
Mfd3'_fwdNEB		GACACATCATGACCGATATCGAGG CGAAGCGATGATCGTCGTCCTGG	For PCR amplification downstream of *mfd*
Mfd3'_revNEB		ATTACGCCAAGCTTGCATGCCTGC AATTGCGCGAGCTGCTCGT	
MfdPCR_F1	PCR of genomic DNA for knockout confirmation	GCTAGCCTGTACGCGTGGCA	Primer set for *mfd* amplification
MfdPCR_R1		CGCGCCTTGACCTTCTTCTCCTG	
MfdqPCRF1	qRT-PCR primer set 1	CATCTGCAGACCTTCACCAA	Primers for the *mfd* gene
MfdqPCRR1		CGTCTTTGAGACCTTCGATCAC	
MfdqPCRF2	qRT-PCR primer set 2	GCTACGCCTACTTCCTGTATC	Primers for the *mfd* gene
MfdqPCRR2		ATCTCCAGATCCTTCATGGC	
PcrAUvrDqPCRF1	qRT-PCR primer set 1	GGCACGGCTACAACTACAA	Primers for *uvrD1* (MSMEG_5534)
PcrAUvrDqPCRR1		CGGATGAACACCTCTTCCAG	
PcrAUvrDqPCRF2	qRT-PCR primer set 2	CTGAACTCGAACTTCTCGATCTAC	Primers for *uvrD1* (MSMEG_5534)
PcrAUvrDqPCRR2		GATCAGCTCGTTCTTGAGGTT	
16s rRNA F	qRT-PCR primer set	GGGAGCGAACAGGATTAGATAC	Primer set for 16s rRNA control
16s rRNA R		CCTTTGAGTTTTAGCCTTGCG	

Table S1. Primers used in this study.

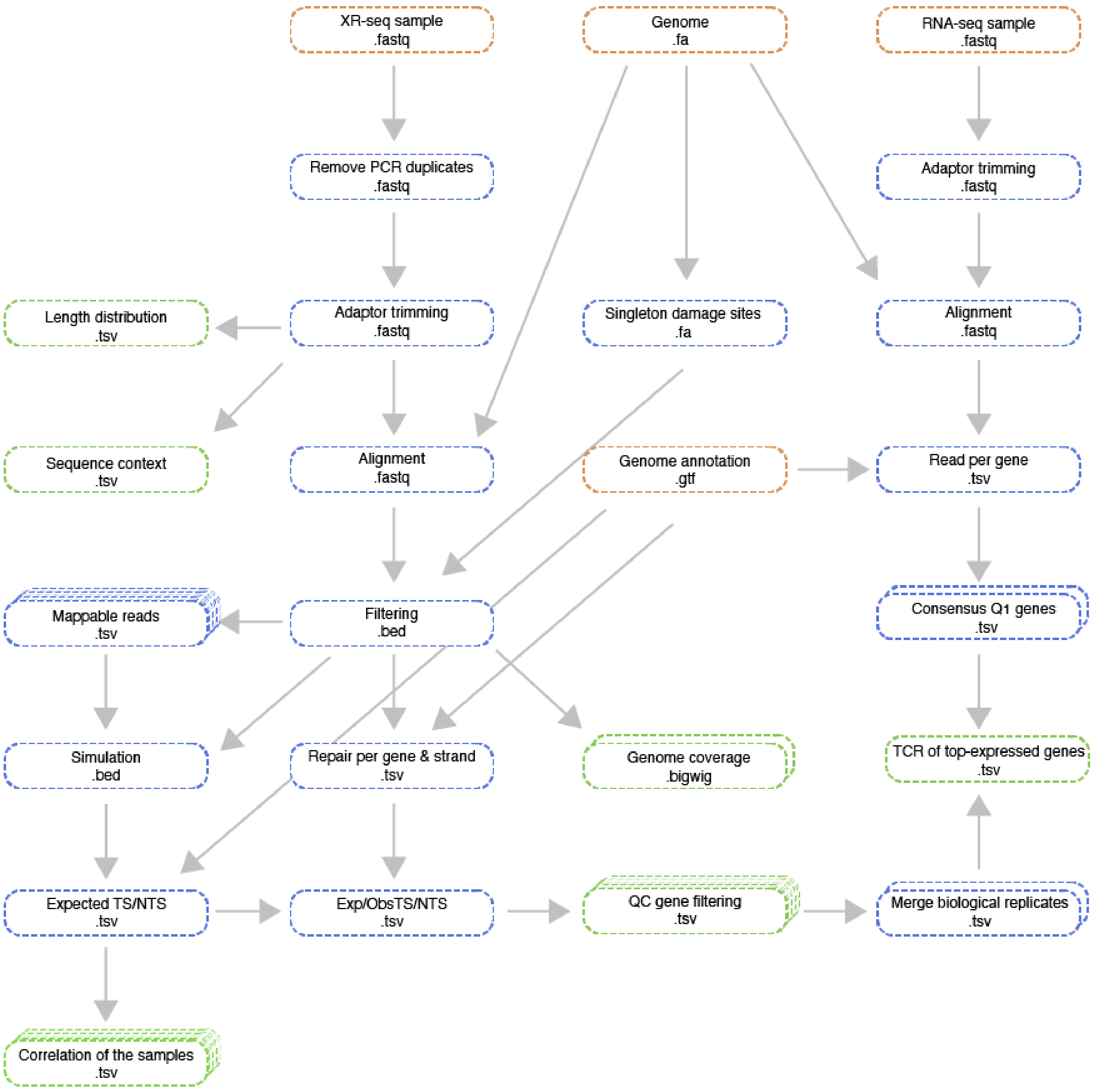

Figure S1. Data analysis workflow. Snakemake was used to allow scalability and reproducibility, and all the custom scripts, commands, and parameters for the publicly available tools can be found in the repository.
